# Transcranial Photobiomodulation to Improve Cognition in Gulf War Illness

**DOI:** 10.3389/fneur.2020.574386

**Published:** 2021-01-21

**Authors:** Paula I. Martin, Linda Chao, Maxine H. Krengel, Michael D. Ho, Megan Yee, Robert Lew, Jeffrey Knight, Michael R. Hamblin, Margaret A. Naeser

**Affiliations:** ^1^VA Boston Healthcare System, Boston, MA, United States; ^2^Department of Neurology, School of Medicine, Boston University, Boston, MA, United States; ^3^Department of Radiology and Biomedical Imaging, University of California, San Francisco, San Francisco, CA, United States; ^4^Department of Biostatistics, School of Public Health, Boston University, Boston, MA, United States; ^5^VA Boston Healthcare System, National Center for Posttraumatic Stress Disorder, Boston, MA, United States; ^6^Wellman Center for Photomedicine, Massachusetts General Hospital, Boston, MA, United States; ^7^Laser Research Center, Faculty of Health Sciences, University of Johannesburg, Johannesburg, South Africa

**Keywords:** photobiomodulation (PBM), light-emitting diode (LED), cognitive function, Gulf war illness (GWI), LLLT (low-level laser/light therapy)

## Abstract

**Introduction:** Approximately 25–30% of veterans deployed to Kuwait, 1990-91, report persistent multi-symptom Gulf War Illness (GWI) likely from neurotoxicant exposures. Photobiomodulation (PBM) in red/near-infrared (NIR) wavelengths is a safe, non-invasive modality shown to help repair hypoxic/stressed cells. Red/NIR wavelengths are absorbed by cytochrome C oxidase in mitochondria, releasing nitric oxide (increasing local vasodilation), and increasing adenosine tri-phosphate production. We investigated whether PBM applied transcranially could improve cognition, and health symptoms in GWI.

**Materials and Methods:** Forty-eight (40 M) participants completed this blinded, randomized, sham-controlled trial using Sham or Real, red/NIR light-emitting diodes (LED) applied transcranially. Fifteen, half-hour transcranial LED (tLED) treatments were twice a week (7.5 weeks, in-office). Goggles worn by participant and assistant maintained blinding for visible red. Pre-/Post- testing was at Entry, 1 week and 1 month post- 15th treatment. Primary outcome measures were neuropsychological (NP) tests; secondary outcomes, Psychosocial Questionnaires, including PTSD.

**Results:** Primary Analyses (all participants), showed improvement for Real vs. Sham, for Digit Span Forwards (*p* < 0.01); and a trend for Trails 4, Number/Letter Sequencing (*p* < 0.10). For secondary outcomes, Real group reported more improvement on the SF-36V Plus, Physical Component Score (*p* < 0.08). Secondary Analyses included only subjects scoring below norm (50%ile) at Entry, on specific NP test/s. Real and Sham improved at 1 week after 15th treatment; however, at 1 month, only those receiving Real improved further: Digit Span Total, Forwards and Backwards; Trails 4, Number/Letter Sequencing; California Verbal Learning Test-II, long delay free recall; Continuous Performance Test-II, False Alarm Rate; and Color-Word Interference, Stroop, Trial 3, Inhibition; Sham group worsened, toward Entry values. Only those with more post-traumatic stress disorder (PTSD) symptomatology at Entry, receiving Real, continued to have additional PTSD reduction at 1 month; Sham regressed.

**Conclusion:** This study was underpowered (*n* = 48), with large heterogeneity at Entry. This likely contributed to significance or trend to significance, for only two of the NP tests (Digit Span Forwards; Trails 4, Number/Letter Sequencing) and only one general health measure, the SF-36V Plus, Physical Component Score. More subjects receiving Real, self-reported increased concentration, relaxation and sleep. Controlled studies with newer, transcranial LED home treatment devices are warranted; this is expected to increase enrollment.

**Clinical Trial Registration:** www.ClinicalTrials.gov, identifier: NCT01782378.

## Introduction

An estimated 25–30% of 700,000 veterans deployed to the Kuwait Theater, 1990-91, have developed a persistent multi-symptom illness related to neurotoxicant exposures (1, 2). To date, treatments developed for Gulf War Illness (GWI) have been insufficient. Interventions addressing underlying pathologies are needed. Photobiomodulation (PBM) is a plausible therapy to address GWI symptoms. PBM consisting of red and near-infrared (NIR) wavelengths of light may impart beneficial effects on mitochondrial and cellular function, immune function, and inflammation, all of which are affected in GWI.

### Gulf War Illness

Gulf War veterans have presented with Chronic Multi-symptom Illness which includes one or more chronic symptoms (lasting >6 months) from at least two of three symptom categories: (1) musculoskeletal (muscle or joint pain, stiffness); (2) mood-cognition; (3) fatigue. Headaches and/or gastro-intestinal problems may also be present (1, 3). Symptoms have been found to worsen over time, consistent with chronic inflammation (2, 4). GWI veterans' symptoms are heterogeneous. However, the persistent presence of impairments can have profound negative effects on veterans' daily functioning.

### Cognitive Dysfunction in Veterans With GWI

Research on cognitive and behavioral changes associated with GWI has been mixed (5). Although GWI symptoms are heterogeneous, a meta-analysis of published studies regarding neuropsychological/cognitive functioning in GWI veterans, has found evidence of significantly decreased performance within three cognitive domains: attention/executive function, learning/memory, and visuospatial skills (5).

### Mitochondrial Dysfunction

Neurotoxicant exposures including low levels of sarin, cyclosarin, smoke, pollutants from oil well-fires, organophosphate pesticides and pyridostigmine bromide pills are thought to be the main causes of symptoms associated with GWI (4, 6, 7). The latter two can cause CNS tissue loss from direct damage to mitochondria, leading to oxidative stress and mitochondrial dysfunction (4, 8–12). Mitochondrial dysfunction occurring in the brain can produce cognitive dysfunction; while in the muscles it causes chronic pain and fatigue (2, 13–16).

### Immunological and Inflammatory Dysfunction in GWI

Immune dysfunction may also be a factor to consider, regarding therapeutics for GWI (4, 17, 18). Georgopoulos and colleagues observed an abnormal synchronous neural interaction pattern in GWI using magnetoencephalography (MEG), reflecting in part, altered brain function in genetically vulnerable individuals (19). Similar immune system disruption occurs in relapsing-remitting multiple sclerosis, Sjogren's syndrome and rheumatoid arthritis. Additionally, blood biomarkers of inflammation - e.g., lymphocytes, monocytes and C-reactive protein were 90% predictive of GWI in a cohort of veterans (20).

### Photobiomodulation (PBM)

PBM therapy is a safe, non-invasive, painless, non-thermal modality based on a strong body of research dating back to the 1960's (21). Light-emitting diode (LED) or low-level laser/light therapy (LLLT), uses either visible red light (600–700 nm) or near-infrared (NIR) wavelengths of light (usually 810, 830, up to 1,100 nm) to repair damaged or dying cells and tissue (22–24).

### Detailed Cellular Effects of PBM

PBM has produced beneficial cellular effects in controlled trials (25). Red/NIR wavelength photons are absorbed within the mitochondrial membrane by the last complex (IV) of the electron transport chain, cytochrome C oxidase (CCO), particularly in hypoxic/stressed cells (26). This promotes increased adenosine tri-phosphate (ATP) production (24, 27) and increased local vasodilation due to release of nitric oxide from CCO. Along with improved mitochondrial and cellular function (23, 24, 28), reduced oxidative damage and reduced inflammation have been reported (29).

Approximately 3% of NIR LLLT photons applied to the head have been observed to penetrate through skin, scalp, bone and dura mater in human cadavers, enough to reach surface brain cortex (30). NIR (808 nm) photons from laser PBM have been detected at 4–5 cm beneath the site of scalp application (31). PBM to the ear canals has been observed to penetrate to the base of the skull in a cadaver and to reach the mesial temporal lobes, altering emotional modulation on a visual attention task (32). PBM applied to points on the ears has also been reported to reduce pain and insomnia (33).

### Effect of Transcranial PBM in Animals

Positive results from application of transcranial PBM (tPBM) have been observed in animal models of traumatic brain injury (TBI) (34, 35); neurodegenerative diseases such as Alzheimer's Disease (AD) (36–39) and Parkinson's Disease (40–42).

### Brain Imaging and Transcranial PBM in Humans

Application of transcranial light-emitting diodes (tLED) in humans has been shown to increase regional cerebral blood flow (rCBF) in normals (43, 44), in depression (45), severe TBI (46), and chronic mild-moderate TBI (47). Cognitive/behavioral improvements have been reported in chronic TBI (47–52), severe depression (45), acute and chronic stroke (53–55), and dementia (56, 57).

Improved functional connectivity on resting-state functional-connectivity MRI scans (rs-fcMRI) has been observed within intrinsic neural networks, including the default mode network (DMN), following tLED treatments in various patient populations. Chao (57) observed increased functional connectivity within the three posterior nodes of the DMN in chronic dementia patients, along with significant improvements in cognition. The dementia patients were treated at home, using a NIR LED device designed to treat only the cortical node areas of the DMN. Case studies have shown improved functional connectivity in the DMN in chronic stroke/aphasia, along with improved language following a series of tLED treatments (55); likewise, improved cognition and functional connectivity in athletes with repetitive head impacts who may develop chronic traumatic encephalopathy (CTE) – e.g., pro-football (51, 58), and ice hockey (59).

Increased functional connectivity has been observed within 1 min during NIR laser application (1 cm diameter, 808 nm, 250 mW, 318 mW/cm^2^) to the right forehead/frontal pole in adults (ages 18–40) (60). Immediate EEG changes have also been shown in healthy adults (ages 61–74) after one, 20-min tLED treatment applied only to cortical node areas of the DMN (61).

Intranasal delivery of PBM also may have beneficial effects. NIR intranasal LED (pulsed at 10 Hz) is hypothesized to deliver photons to the olfactory bulbs (on orbito-frontal cortex), where olfactory inputs activate medial entorhinal cortex via the hippocampus (62). In a small study, five dementia patients who used a NIR intranasal, pulsed at 10 Hz, daily for 12 weeks (with once weekly, headframe NIR tLED treatments to nodes of the DMN), improved significantly in cognition (56). Four dementia patients using NIR intranasal plus tLED at home, three times per week for 12 weeks, also observed cognitive improvement (57). Application of red intranasal PBM has been observed to improve blood rheology (blood viscosity, plasma viscosity, redox viscosity and red blood cell aggregation), and improve cholesterol levels (blood lipid total cholesterol and low/high-density lipoprotein cholesterol) in patients with coronary heart disease and cerebral infarction (63, 64).

Some benefits for GWI have been found from exercise, cognitive-behavioral therapies, co-enzyme Q10 supplements and individualized acupuncture treatments (9, 65). Two GWI case reports showed symptom reduction in the mood-cognitive domain, pain, sleep and fatigue after a 12-week, at-home transcranial plus intranasal NIR LED treatment series, treating every other day (66).

PBM offers a potentially safe and effective treatment option for veterans with GWI. This current study reports the results of a sham-controlled clinical trial that examined whether a 7.5-week series of red/NIR tLED treatments (plus LEDs applied over the ears, and intranasal LED in each nostril simultaneously) could improve cognition and other health symptoms in veterans with GWI.

## Methods

### Participants

Participants were recruited from subjects in a Department of Defense (DoD) study of a longitudinal cohort of Gulf War Veterans who returned from their deployment to Kuwait (1990-91) through Fort Devens, MA. This cohort has been followed at multiple time points since the end of the GW (67); and through the VA Informatics and Computing Infrastructure/Corporate Data Warehouse (VINCI/CDW) database, with approval from the Veteran's Health Administration (VHA) National Data System (NDS). The San Francisco VA Medical Center (SF VAMC) was a second site on this study (36 Veterans participated in Boston, and 12, in San Francisco on the same protocol). Those recruited from the VINCI/CDW database, resided within a 25-mile radius of the VA Boston Healthcare System (VABHS) or the SF VAMC. The Institutional Review Board at the VABHS and the SF VAMC (University of California, San Francisco) approved the study. In accordance with the Declaration of Helsinki, Informed Consent and HIPAA authorization were obtained. The ClinicalTrials.gov reference is NCT01782378.

### Inclusion Criteria

Participants answered “Yes” during a telephone screening regarding: (1) Difficulty concentrating; and/or (2) Difficulty remembering recent information. Additional inclusion criteria were the following: Must be a veteran deployed in the 1990-91 Gulf War, to the Kuwait Theater; meets criteria for GWI as defined by “Symptom Questions used to identify Gulf War Illness by Kansas Case Definition, and Chronic Multi-symptom Illness by Fukuda Case Definition” (1, 3) – i.e., must have presence of one or more chronic symptoms (lasting >6 months) from at least two of three symptom categories from Fukuda 1998: (1) musculoskeletal (muscle pain, or joint pain, stiffness); (2) mood/cognition; (3) fatigue. Participants filled out the symptom questionnaire (3) at Entry to determine if they met these criteria. The veterans must have exhibited symptoms after deployment (with exposures) and have current symptomatology as noted above, fulfilling the Kansas Case Definition and Chronic Multi-symptom Illness definition of GWI.

In addition, the age range was 38–65 years; must be physically able to travel to the VABHS, Jamaica Plain campus or SF VAMC for Neuropsychological (NP) testing and tLED treatments; and must meet screening criteria from the Eligibility Screening, NP tests (administered at in-office Screening visit): Trail Making Test A & B (68, 69); Controlled Oral Word Association Test [COWAT (FAS) (70, 71); California Verbal Learning Test (CVLT) - II (72); Color-Word Interference Test, Stroop (73). For inclusion, participants were required to score at least 2 SD below the standardized norm (age, education) on at least one of the above-mentioned NP screening tests, or 1 SD below the standardized norm on at least two of them.

In addition, the Word Reading Subtest from the Wide Range Achievement Test-4 (74) was used to estimate premorbid level of cognitive functioning. The SD for each participant on each NP screening test was adjusted by his/her estimated premorbid cognitive level. A symptom validity measure, the Test of Memory Malingering (75) was also administered at screening. Participants who failed Trial 2, or Trial 1 and 2 were excluded from the study. If a participant failed Trial 1, but did not fail Trial 2, he/she was not excluded if he/she showed evidence of poor learning on other NP screening tests, such as the CVLT (76).

Additional Screening questionnaires were administered: Short Form McGill Pain Questionnaire (77); VAS pain rating (0–10); and the PTSD Checklist- Civilian (PCL-C) (78). Participants were included only if the VAS pain rating was ≤ 7/10, and ≤ 38/50 on the Short Form McGill Pain Questionnaire, as pain has been shown to influence cognition (79).

### Exclusion Criteria

Exclusion criteria were the following: Less than age 38, or greater than age 65; presence of a neurodegenerative disease such as Amyotrophic Lateral Sclerosis, Parkinson's Disease, dementia; presence of a life-threatening disease such as cancer; presence of a severe mental disorder such as schizophrenia, or bipolar depression (not associated with PTSD); current substance abuse or active treatment within the last 6 months.

### Randomization

**Figure 1:**
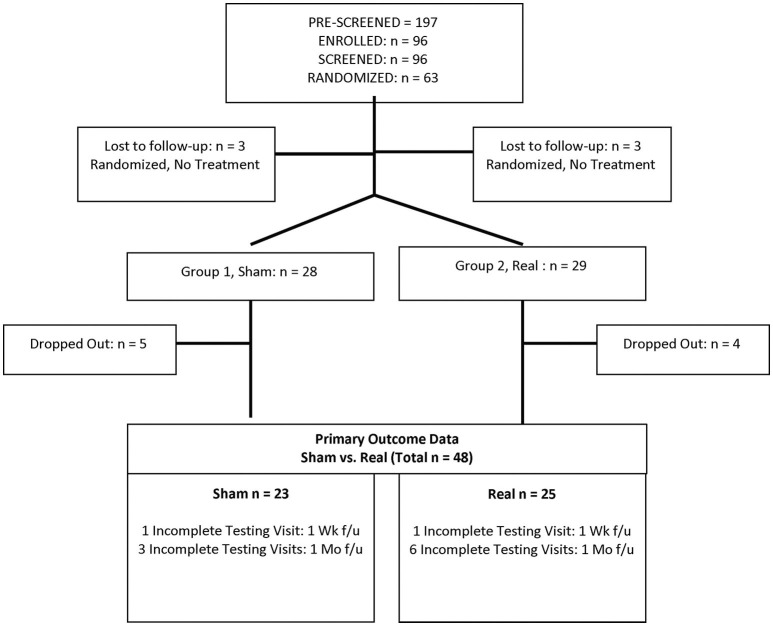
Study consort chart (VA Boston healthcare system and San Francisco VA medical center).

A total of 63 eligible participants were randomized to the Sham or Real LED treatment groups (computer-assigned randomization with GraphPad.com, in blocks of 10). Three in each group dropped out before any treatment visits; and nine of the remaining 57 who started treatment visits dropped out (five from Sham, and four from Real) without any Post- testing, leaving a total of 48 participants (Sham group, 23 participants; Real group, 25 participants). See Consort Chart, Figure 1. Only the study coordinator had the code for the groups.

The demographics for each group (Sham and Real) are provided in Table 1; there were no significant differences (Pearson Chi-Square) between the two groups on any of the demographic variables.

**Table 1:** Demographics and clinical characteristics of veterans with GWI.

	**Group 1: Sham LED**	**Group 2: Real LED**		**Pearson chi-square**
	**Tx**	**Tx**	**All veterans**	
*N*	23	25	48	
Age (years, SD)	52.4 (6.6)	51.9 (5.6)	52 (6)	0.799
Education (years, SD)	14.2 (1.9)	14.2 (1.99)	14 (1.9)	0.601
No. females (%)	2 (9%)	6 (24%)	8 (17%)	0.155
No. with TBI	9 (40%)	7 (28%)	16 (33%)	0.372
No. with blast exposure	5 (22%)	3 (12%)	8 (17%)	0.357
No. with PCL-C >36 at entry	13 (56%)	19 (76%)	32 (66%)	0.085
No. with BDI >30 (severe depression)	2 (8%)	6 (26%)	8 (16%)	0.178
No. on pain medications^*^	5 (22%)	3 (12%)	8 (17%)	0.223
No. on sleep medications^*^	3 (13%)	1 (4%)	4 (8%)	0.190
CMI criteria (3)	All endorsed at least 2/3 CMI criteria at entry			
Memory/concentration difficulties	All endorsed memory and/or attentional difficulties at entry			
Region served	All served in Kuwait Theater			

*Mean (SD) or N (%), reported*.

*CMI, Chronic Multi-symptom Illness; TBI, traumatic brain injury; PCL-C, Post Traumatic Stress Disorder (PTSD) Checklist for Civilians; BDI, Beck Depression Inventory*.

* *Pain medications and Sleep medications were not recorded for 8 participants*.

### Blinding

Each subject was assigned a random ID number and blinding was maintained by the study coordinator throughout the study. None of the other staff were aware of group assignment. This included the Neuropsychologist performing the Pre-/Post- testing, the principal investigator, the participant and family members, as well as those performing the LED treatments (Sham or Real), explained further under the subsection, Blinding at Each LED Treatment Session.

### NP Tests and Questionnaires, Pre-/Post- Sham or Real LED Intervention Series

The NP testing was performed within 1 week before the intervention series, at 1 week after completion, and at 1 month later. Each NP Testing session was completed in 1.5 h. The NP tests (primary outcome measures), and the Psychosocial, Self-Report Measures/Questionnaires (secondary outcome measures) are listed in Table 2.

**Table 2:** a,b. Primary outcome measures, neuropsychological tests and secondary outcome measures, psychosocial and health questionnaires.

**a**.		
**Primary outcome measures**		
**17 Neuropsychological domain/individual tests and subtests**		
**Attention/executive function**		
**Digit span (WAIS-V)** **(****80****)**		
Forward		
Backwards		
Total: forward + backward		
**Delis-Kaplan executive function test (DKEF) trails** **(****73****)**		
Trails 2		
Trails 4		
**Color-Word interference (Stroop)** **(****73****)**		
Trial 3		
Trial 4		
**Verbal memory and learning**		
**California verbal learning test II (CVLT-II)** **(****72**)^*^		
Total trials 1–5 (learning)		
Short delay free recall		
Short delay cued recall		
Long delay (20 Min) free recall		
Long delay (20 Min) cued recall		
**Connor's continuous performance test II (CPT)** **(****81**, **82****)**		
Mean RT for % correct detections		
% False alarms		
Detectability (d')		
**Visuospatial/memory**		
**Rey osterrieth complex figure test (ROCFT)** **(****83****–****85****)**		
Immediate recall		
Delayed recall		
**b**.		
**Secondary outcome measures**		
**46 psychosocial and health questionnaires/self reports and subscores**		
**PCL-C PTSD checklist civilian** **(****78****)**		
**Visual analog pain rating scale (VAS)** **(****86****)**		
**Short form McGill pain questionnaire** **(****77****)**		
**West Haven-Yale Multidimensional Pain Inventory (WHYMPI)** **(****87****)**		
**Part A**	**Part B**	**Part C**
Interference Support Pain severity Life-control Affective disorders	Negative responses Solicitous responses Distracting responses	Household chores Outdoor work Activities away Social activities General activity
**Multi-dimensional fatigue inventory** **(****88****)**		
General fatigue		
Physical fatigue		
Reduced activity		
Reduced motivation		
Mental fatigue		
**Beck Depression Inventory II (BDI-II)** **(****89****)**		
**Health Symptom Checklist (HSC)** **(****67****)** (raw score and frequency score for each domain)		
General health		
Musculoskeletal pain		
Mood		
Cognitive		
Gastrointestinal		
Fatigue/sleep		
**Veterans RAND 36 item health survey, SF-36V Plus** **(****90**, **91**)^**^		
Physical functioning Role limitations due to Phys. Fct. Bodily pain General health Vitality		Social functioning Role limitations due to Emot. and mental Health problems Mental health ^†^Physical composite score ^†^Mental composite score
**Pittsburgh Sleep Quality Index (PSQI)** **(****92****)**		
Global PSQI score		
**Epsworth sleepiness scale** **(****93****)**		
**Karolinska sleepiness scale** **(****94****)**		

* *Alternate Version administered at every other testing session to avoid practice effects*.

** *Developed by RAND as part of the Medical Outcome Survey Study*.

† *Represents a combination of the other HSC subtests within the domains of mental health or physical health*.

### NP Tests, Domain Scores

Domain Scores were also calculated as a composite score to represent different areas of cognition: Attention and Executive Function (AF); Learning and Memory (LM); and Psychomotor/Visual Spatial (P/VS). AF included 8 subtests: Digit Span Total; Trail-Making Test conditions 2 and 4; reaction time for correct detections, false alarm rate and delta prime measures on the Continuous Performance Test (CPT); and Color-Word Interference Test, conditions 3 and 4. LM included 7 subtests: CVLT-II, total; short and long delay - free and cued recall; and Rey-Osterreith Complex Figure Test (ROCFT), immediate and delayed recall. P/VS included 2 subtests: CPT, reaction time for correct detections; and the ROCFT, copy.

### LED Intervention Series (Sham or Real)

#### Schedule of Visits

The half-hour LED treatments (Sham or Real) were scheduled for twice a week, with at least 48 h between treatments, for 7.5 weeks (15 treatments).

#### Real LED Devices

**Figure 2:**
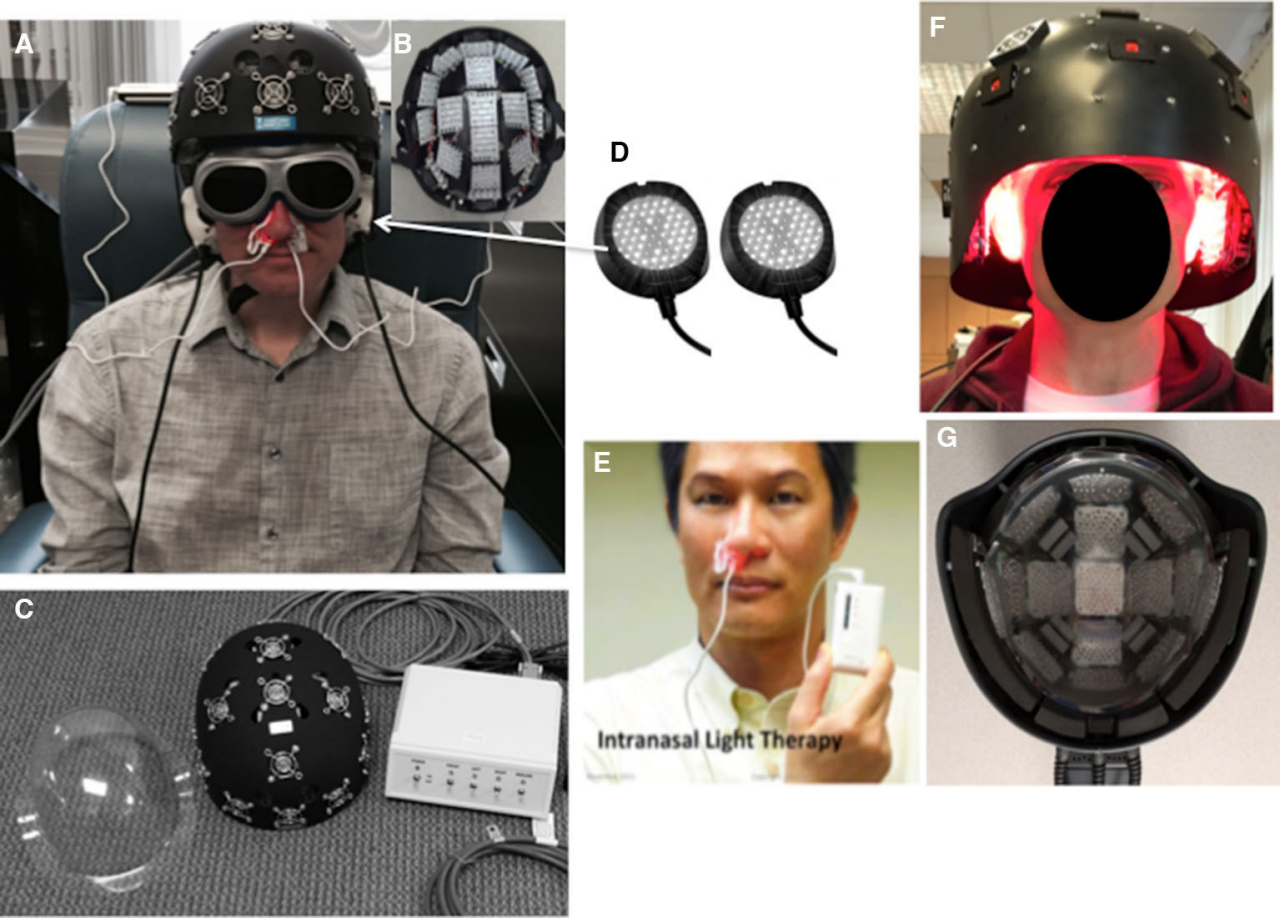
LED devices. **(A)** NIR, LED-lined helmet (PhotoMedex, Horsham, PA). **(B)** LED cluster head placement configuration inside PhotoMedex helmet. **(C)** Separate clear plastic liner assigned to each participant to fit inside the PhotoMedex helmet (to protect diodes and for hygiene), and control box where each row could be turned on and off separately. **(D)** Two NIR, LED cluster heads for placement over the ears (MedX Health, Toronto). **(E)** Red intranasal LED applicator (Vielight, Toronto; Hayward, CA); note, an identical NIR intranasal was also used in the other nostril. **(F)** NIR, LED-lined helmet (Thor Photomedicine, London, UK; Hampstead, MD), used if the participant's head had a circumference greater than 24 inches. **(G)** LED cluster head placement configuration inside the Thor helmet, with both red and NIR diodes in each cluster head. Only the NIR diodes were turned on for this study. During all helmet treatments, the midline row was first treated, then turned off, and then the left and right sides were treated. The fans for each LED cluster head inside the helmet (for cooling) were always turned on. Sham and real treatments felt identical. Participants and assistant wore goggles to block the red intranasal light. NIR, near-infrared wavelength; LED, light-emitting diodes. See Table 3 for LED specifications and treatment parameters. **(E)** Printed with permission from the Vielight Co.

Treatments were administered using three types of LED devices, simultaneously at each visit – this included (1) an LED-lined helmet (Photomedex or Thor); (2) LED cluster heads for ear placements (except when the larger, Thor helmet was used which covered the ears); and (3) two intranasal LED applicators. All participants received treatment with all three types of devices applied at the same time during each treatment, as described below. The Real LED devices are shown in Figure 2. The LED parameters and equipment specifications for each Real LED device are provided in Table 3. The VABHS Safety Subcommittee of the IRB approved all LED devices.

**Table 3:** Real LED equipment parameters and treatment specifications.

	**PhotoMedex LED lined helmet.** **Each cluster head**	**MedX health LED cluster heads, 1 on each ear.** **Each cluster head**	**Vielight red, 633 nm intranasal LED device**	**Vielight NIR, 810 nm intranasal LED device**	Thor photomedicine LED lined helmet^**^ Midline clusters turned on, only **Set A**	**Thor photomedicine** LED lined helmet^**^ **left and right sides turned on, same time** **Set B**
Cluster head size	4.5 cm ×4.8 cm (21.6 cm^2^)	2 inch diameter (22.48 cm^2^)	1 cm^2^	1 cm^2^	6.3 cm diameter (28.3 cm^2^)	6.3 cm diameter (28.3 cm^2^)
Number of cluster heads	18	2	Single diode	Single diode	5	10
No. of diodes per cluster head	20	52	1	1	69	69
Wavelength, nm	830	870	633	810	34–660; 35–850	34–660; 35–850
Power output, mW per cluster head	626	90	8	14.2	1,265.6	1,075.4
Power density, mW/cm^2^	29	4	8	14.2	41	35
Pulse frequency, Hz	CW	CW	CW	10	CW	CW
Pulse duty cycle, %	–	–	–	50	–	–
Total time per session	28 min 10 s^*^	4 min	25 min	25 min	10 min 42 s	12 min 36 s^***^
Dose: energy density, J/cm^2^	26	1	12	10.65	26	26
Joules	1,058	21.6	12	10.65	812.5	813
Total joules per device	19,043	43.2	12	10.65	4,063	8,130

* *Includes two sequential placement sets with nominal power output per cluster head, 626 mW: First placement set, midline only, 6 cluster heads turned on (14 min, 5 sec); Second placement set, Left and Right sides of head simultaneously, total 12 LED cluster heads turned on (14 min, 5 s). When PhotoMedex helmet, and two LED cluster heads over the ears, and two intranasals were used, Joules per treatment = 19.1 kJ, Total Joules after 15 Treatments = 286.6 kJ*.

** *Used with participants where the head circumference was >24 inches*.

*** *Two sequential Placement Sets: Total Time, Set A plus Set B, was 23 min 18 sec. Note, the power output and power density varied slightly, for the different number of cluster heads turned on at the same time, 5 (Set A), or 10 (Set B). When Thor helmet, and two intranasals were used, Joules per Treatment = 12.2 kJ, Total Joules after 15 Treatments = 183.2 kJ*.

*CW, Continuous Wave*.

#### Real LED Device 1, LED-Lined Helmet

A Real, NIR LED-lined helmet was manufactured by PhotoMedex, Inc., Horsham, PA (Figures 2A,B); or by Thor Photomedicine, Inc., London, UK; Hampstead, MD (Figures 2F,G). If a participant's head circumference was larger than 24 inches, the larger LED-lined helmet (Thor Photomedicine helmet) was used. Each LED-lined helmet contained previously-approved, FDA-cleared LED cluster heads which were considered non-significant risk. Each participant wore a clear plastic helmet liner (assigned to that participant only) to protect the LEDs from hair, and for hygiene reasons (Figure 2C).

#### Real LED Device 2, LED Cluster Heads for Ear Placements

Two NIR LED cluster heads (each, 2-inch diameter) were manufactured by MedX Health, Mississauga, ON, Canada (Figure 2D). The smaller, Photomedex helmet did not cover the ears, thus these LEDs were used simultaneously on the left and right ears for 4 min, toward the end of each treatment visit when the Photomedex helmet was used. They were lower in power (90 mW) than the previously-approved, FDA-cleared (500 mW), non-significant risk MedX Health LED cluster heads. The larger, Thor Photomedicine LED Helmet contained LEDs that covered the ears.

#### Real LED Device 3, Intranasal LED Applicators

Two intranasal LEDs (red and NIR single-diode applicators) were manufactured by Vielight, Inc., Toronto, Canada; Hayward, CA. One intranasal LED was placed into each nostril, held in place with a plastic clip. Placement of the red intranasal LED (Figure 2E), and the NIR intranasal LED (not shown) was alternated by side (left/right), at each session. Due to the very low power of each intranasal LED, 8 mW and 14.2 mW (red and NIR, respectively), each was considered to be within the low-risk device, FDA category of General Wellness, no medical claims made.

#### Sham LED Devices

Sham LED devices that looked and felt identical to the Real LED devices were also manufactured by each LED company listed above. No red or NIR photons were emitted from any of the Sham LED devices. The fans in a LED-lined Sham helmet were turned on during the entire half-hour Sham LED treatment session, as was also done during a Real LED session. The helmet fans were used for cooling.

#### Blinding at Each LED Treatment Session (Sham or Real)

For the purpose of blinding during a treatment session (Sham or Real), the participant and the assistant administering the treatment wore goggles (Diode Laser Safety Goggles – Model 55, LS-DIO-55, Phillips Safety Products, Inc., Middlesex, NJ). The goggles blocked 600–900 nm wavelengths, used particularly to block visual perception of the red, 633 nm photons emitted from the red intranasal diode. The NIR photons are beyond the visible spectrum. Two LED treatment rooms were used; the study coordinator instructed the assistant regarding which treatment room to use for each LED treatment (The LED equipment could also be moved randomly, from one treatment room to another). All LED devices looked and felt identical (Sham and Real).

To address the possible effect of skin pigmentation on photon absorption, the Fitzpatrick Skin Type Scale (95) was used to determine skin type (pigmentation level), ranging from 1 (very fair skin) to 6 (darkest skin). The dose was adjusted when treating participants who had the lightest pigmentation, 1; or the darkest pigmentation, 6. Four participants had levels 1 or 6, and all others, in between. For those with the lightest skin, the treatment time/dose was reduced by 25%, and for those with the darkest, increased by 25%.

#### Possible Changes in Medications During Participation

Participants were encouraged not to change medications or dosages, if possible, during participation in the study. At each treatment visit (twice a week), they answered questions regarding possible changes in medications, sleep, pain or other changes/complaints since the previous treatment.

#### Treatment Compliance and Safety

Forty-eight participants completed all 15 treatments, however, only 25% of group 1 (Sham) and 21% of group 2 (Real) adhered to the intervention schedule of in-office visits twice per week for 7.5 weeks. If a participant missed a treatment, that treatment was added at the end of the series. Participants were withdrawn from the study if they had only one treatment within a 2-week period. An average total of 8.1 weeks was required for participants to complete 15 treatments (mean 8.35, Sham; mean 7.9, Real). Four participants required > 10 weeks to complete the 15 treatments (3 Sham; 1 Real). Nine of the 57 participants who began treatments dropped out before any Post- testing was performed (15.7%). There were no adverse events or negative side effects.

### Statistical Analyses

Primary Analyses described below were performed on data for all 48 participants. Secondary Analyses described below were performed on data for only those participants who scored below norm at Entry, on a specific NP test. Not all participants scored below average on all NP tests at Entry due to large heterogeneity among scores at Entry.

### Primary Analyses (All Participants)

Each NP test was modeled as a two-factor ANOVA with factors, time (Pre-Tx Entry, 1 week Post- Tx, and 1 month Post- Tx) and treatment (Sham, Real). Without correction for multiple comparisons, each test outcome was first tested as a fixed effects model. The fixed effects model was re-run adjusting for baseline demographic factors. These additional analyses showed little effect on the results and are not shown.

We recruited 63 participants, six dropped out, and 57 began treatments. However, nine cases had only Entry/baseline data; hence, no subsequent measurements of response to treatment. The 48 remaining cases provided little statistical power, even without correction for multiple comparisons. Besides having *p* <5% indicate potentially useful predictors, we also displayed factors with a *p*-value between 5 and 10%, which might also prove useful predictors for future studies. We designated as potentially useful, those factors falling between 5 and 10%, with the smallest alternative *p*-values. We re-ran the fixed effects ANOVA model, adding the assumption that the factor “subject” was a random effect. This gave us a random effects *p*-value and a fixed effects *p*-value for each factor.

In summary, our method used a set of two *p*-values to identify potentially useful factors; a measure of relative consistency rather than an absolute measure of significance. We kept the threshold small, p <10%, out of respect for the level of Type I error.

Primary Analyses for the secondary outcome measures (Psychosocial, Self-Report Measures/Questionnaires) were performed in the same manner. Baseline demographic factors did not affect the results. All analyses were run using SAS version 9.4.

### Secondary Analyses (Only Participants Who Scored Below Norm at Entry, on a Specific Test)

As mentioned, there was large heterogeneity within the scores for the cognitive tests at Entry, and not all participants had scores that were below the norm on all NP tests. This is consistent with the known heterogeneity of GWI symptomology. Therefore, for a single NP test, the data for only those participants who scored at, or below, a z-score of −0.5 (below 50th percentile for the norm) on that specific NP test at Entry, were included in the Secondary Analyses. This resulted in a varied number of subjects for each NP test, reducing the power due to fewer participants per group. Every participant was included for some of the NP tests. A fixed effects ANOVA model and a mixed-design random effects analysis with the same factors as for the Primary Analyses, were performed for each separate NP test.

For the secondary outcome measures/Questionnaires, cutoff scores were used to classify participants. On the PCL-C, analyses were completed separately for those who scored <36 at Entry (mild or no PTSD symptomatology), and for those who scored >36 at Entry (evidence of PTSD symptomatology) (96, 97). For the Beck Depression Inventory (BDI) (89, 98), analyses were completed for subgroups based on Entry scores: Little to no depression, scores of 0–12; mild depression, 13–19; moderate, 20–29; and severe, >30. Analyses were completed using SPSS v.26.

### Intent-to-Treat (ITT) Analyses

Of 63 participants enrolled, six dropped out before treatments, and nine only completed a few treatments; never returning for evaluation of treatment effects (Post- testing). Excluding these nine participants without Post- testing, constitutes modified ITT (99). The modified ITT analysis covers the 48 remaining participants. Dropouts per group (Sham and Real) were approximately equivalent. See Consort Chart, Figure 1. We included only those remaining 48 subjects with Pre- and at least most Post- testing data, because our goal was to explore the data to detect measures sensitive to treatments effects.

## Results

### Primary Analyses (All Participants)

#### Primary Outcome Measures

**Figure 3:**
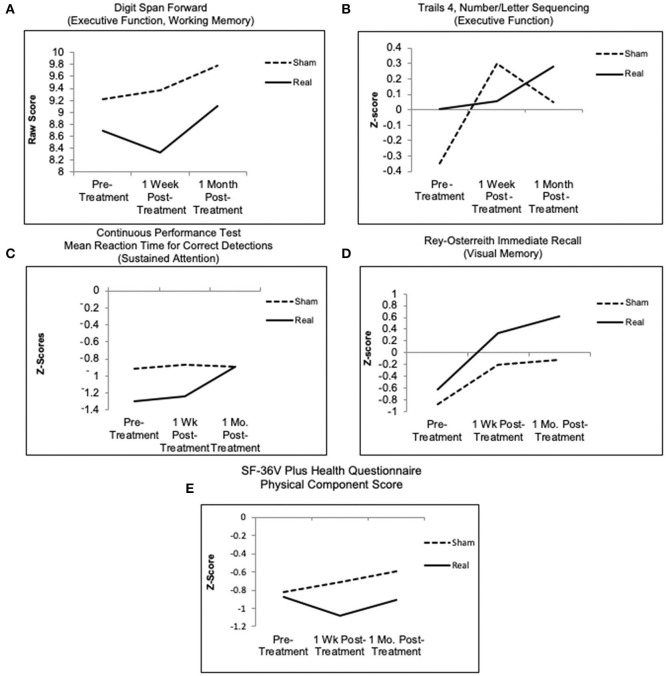
Graphed results for primary analyses (all participants), mixed design random effects model for primary outcome measures/cognitive measures, for four NP tests over time. **(A,B,D)** Higher scores indicate better outcome. **(C)** Continuous performance test, only the real group improved, showing shorter reaction time at 1 month post- treatment; the sham group showed no change. **(E)** SF-36V plus health questionnaire, physical component score (a secondary outcome measure), only the real group endorsed fewer symptoms at 1 week post- LED, relative to entry; whereas the sham group endorsed more symptoms at 1 week and 1 month. See Tables 4A,B for p-values.

The *p*-values for Sham compared to Real for the primary outcome measures are shown in Table 4a (fixed effects results shown only for *p* < 0.10). Only one primary outcome measure showed significant improvement at the *p* < 0.10 level, on the mixed design random effects model analysis in favor of Real compared to Sham over time, Digit Span Forwards (Working Memory, Executive Function), *p* < 0.01, Est Value −0.55, Std Err 0.33. A trend to significance was present for Trails 4, Number/Letter Sequencing (Executive Function), *p* < 0.10, Est. Value −0.36, Std Err 0.22. See Figures 3A,B.

**Table 4a:** Primary analyses (all participants), for fixed effects model and mixed design random effects model, for primary outcome measures/cognitive measures.

**NP test list**	**Mixed design random effects model**				**Fixed effects model** ***p*** **<** **0.10**			
**Cognitive measures**	***p*-value**	**Estimated value**	**Std Err**		***p*-value**	**Estimated value**	**Std Err**	
**Results** ***p*** **<** **0.05**								
**Digit span**								
Forward	0.01	−0.55	0.33	Raw	0.05	−0.59	0.32	Raw
**Results between** ***p*** **>** **0.05 and** ***p*** **<** **0.10**								
**DKEF trails**								
Trails 4	0.10	−0.36	0.22	z-score	0.10	−0.31	0.19	z-score
**Results** ***p*** **>** **0.10**								
**Digit span**								
Backwards	0.88	−0.13	0.88	Raw				
Total F&B	0.81	−0.14	0.59	Raw				
	0.99	−0.14	0.59	z-score				
**DKEF trails**								
Trails 2	0.49	−0.13	0.19	z-score				
**Color word interference (Stroop)**								
Trial 3 (inhibition)	0.48	0.16	0.23	z-score				
Trial 4 (inhibition/switching)	0.30	−0.18	0.17	z-score				
**CVLT II, alternating versions**								
Total trials 1–5	0.60	−0.12	0.23	z-score				
Short delay free recall (SDFR)	0.25	−0.32	0.27	z-score				
Short delay cued recall (SDCR)	0.46	0.21	0.28	z-score				
Long delay free recall (LDFR)	0.76	−0.08	0.25	z-score				
Long delay cued recall (LDCR)	0.87	0.05	0.31	z-score				
**Continuous performance test-II**								
Mean RT for % correct detections	0.18	−0.21	0.16	z-score	0.07	−0.24	0.13	z-score
% False alarms	0.44	0.16	0.20	z-score	0.06	0.29	0.15	z-score
Detectibility (d')	0.45	−1.30	1.70	Raw	0.01	1.37	0.48	Raw
**Rey osterrieth complex figure test**								
Immediate recall	0.18	−0.21	0.16	z-score				
Delayed recall	0.44	0.16	0.20	z-score				

Additionally, Figures 3C,D shows the Real group improved on the CPT, mean reaction time for correct detections (Sustained Attention), while the Sham group remained flat; and both groups improved on the Rey-Osterreith Complex Figure Test (ROCFT), immediate recall (Visual Memory), with a trend toward greater improvement for the Real group. These two NP tests, however, and other cognitive measures did not show significance between the groups.

#### Secondary Outcome Measures

The *p*-values for Sham compared to Real for the secondary outcome measures, Psychosocial, Self-Report Measures/Questionnaires are shown in Table 4b (fixed effects results shown only for *p* < 0.10). On only one Secondary Outcome measure, the SF-36V Plus - Physical Component Score (PCS), those in the Real group endorsed fewer physical complaints over time, compared to those in the Sham group, *p* < 0.08, Est Value −0.31, Std Err 0.16. See Figure 3E. A trend to significance was present for Short Form McGill Pain Questionnaire, *p* < 0.08, Est. Value 3.36, Std Err 1.86. No change was observed in the Real group, while there was a slight decrease in pain in the Sham group. No differences were found for other secondary outcome measures including fatigue, sleep, or mood.

**Table 4b:** Primary analyses (all participants), for fixed effects model and mixed design random effects model, for secondary outcome measures/psychosocial self-report measures.

	**Mixed design random effects model**				**Fixed effects model** ***p*** **<** **0.10**			
**Psychosocial self-report measures**	***p*-value**	**Estimated value**	**Std Err**		***p*-value**	**Estimated value**	**Std Err**	
**Results between** ***p*** **>** **0.05 and** ***p*** **<** **0.10**								
**SF-36V Plus**								
Physical component score (PCS)	0.08	−0.31	0.16	z-scores	0.02	−0.32	0.16	z-scores
Short form McGill pain questionnaire	0.08	3.36	1.86		0.09	3.36	1.86	
**Results** ***p*** **>** **0.10**								
VAS pain rating	0.15	0.91	0.62					
**Multi-dimensional fatigue inventory**								
General fatigue (GF)	0.82	−0.21	0.88					
Physical fatigue (PF)	0.20	1.12	0.84		0.07	1.04	0.57	
Reduced activity (RA)	0.71	−0.32	0.86					
Reduced motivation (RM)	0.85	0.14	0.73					
Mental fatigue (MF)	0.44	−0.73	0.94					
Beck depression inventory II	0.82	0.38	1.63					
PCL-C (PTSD checklist, civilian)	0.43	−1.98	2.53					
**SF-36V plus**								
Mental component Score (MCS)	0.36	0.17	0.20	z-scores				
**Pittsburgh sleep quality index**								
Global PSQI score	0.70	0.32	0.83					
Karolinska sleepiness scale (KSS)	0.48	0.36	0.50		0.06	0.71	0.37	

### Secondary Analyses (Only Participants Who Scored Below Norm at Entry, on a Specific Test)

#### Primary Outcome Measures

**Figure 4:**
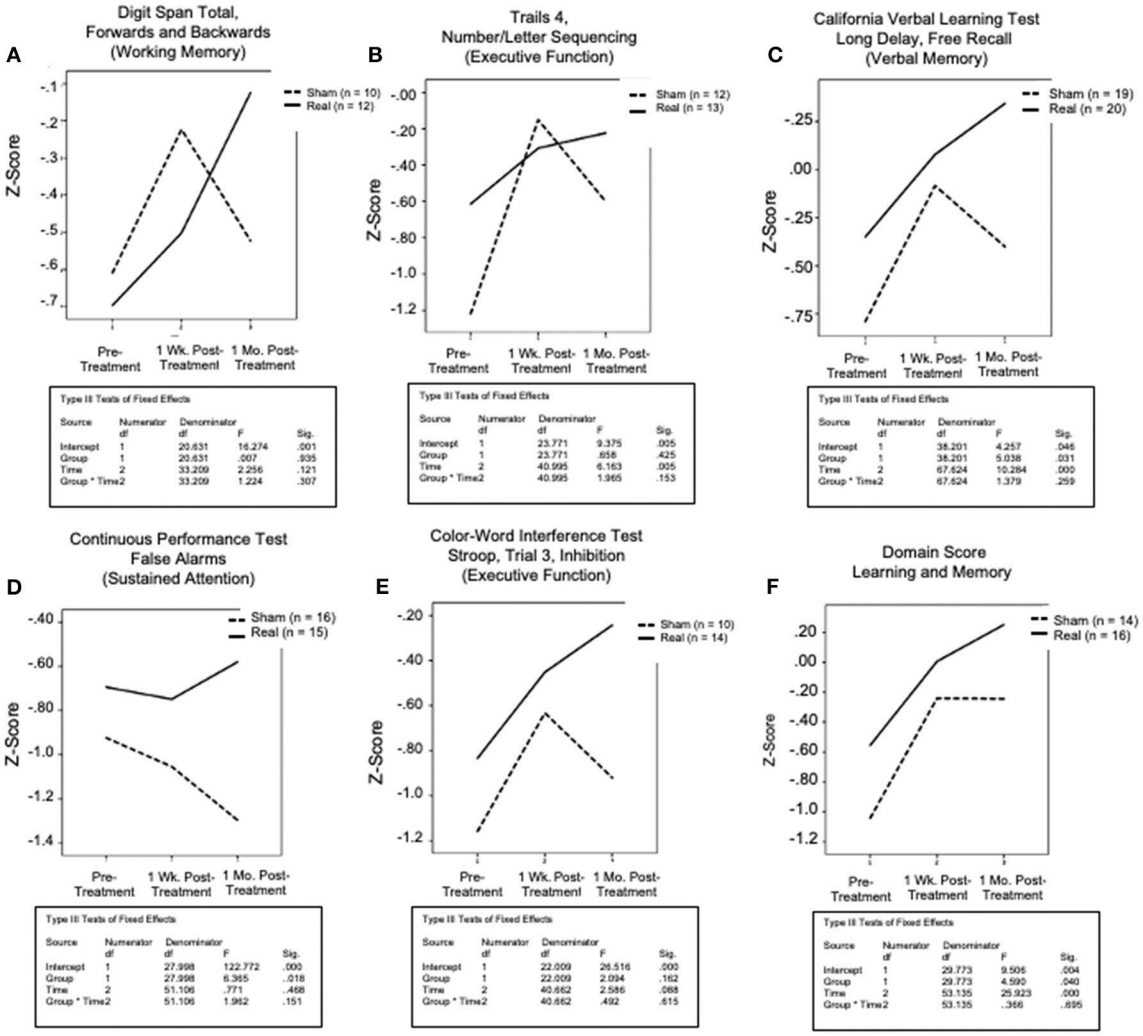
Graphed results for secondary analyses (only subjects who scored below norm at entry, on a specific test), primary outcome/cognitive measures for five NP tests and one domain score. These reflect the same factors as the primary analyses [time (pre-Tx entry, 1-Wk post- Tx, 1-Mo post- Tx); and treatment (sham, real), and their interaction], for each. The domain score for learning and memory reflects a composite score for seven NP tests: the CVLT (total; short and long delay - free and cued recall); and the rey-osterreith complex figure test (immediate and delayed recall). There was a consistent pattern across all graphs, where both groups showed improvement at 1 week post- the final, sham or real LED treatment, but only those who received real LED continued to improve at the 1-month, post- testing time point. Those who received sham, regressed at 1 month, approaching entry values.

After filtering to include only participants whose scores were below norm (50%ile) at Entry, on a specific NP test, a distinct, similar pattern of results was observed for the primary outcome/cognitive measures – i.e., both the Sham LED group and the Real LED group improved at 1 week after the final, 15th LED treatment, suggesting a placebo effect. However, at 1 month after the final, 15th LED treatment, only those in the Real LED group continued to improve, while those in the Sham LED group worsened, and their NP test scores returned toward Entry values. See Figures 4A–F. There was no significant group x time interaction.

The five cognitive tests that showed continued, increased performance at 1 month after the final, 15th Real LED treatment included: (1) Digit Span Total, Forwards and Backwards (Working Memory, Executive Function); (2) Trails 4, Number/Letter Sequencing (Executive Function); (3) CVLT-II, long-delay, free recall (Verbal Memory); (4) CPT, False Alarm Rate (Sustained Attention); and (5) Color-Word Interference Test, Stroop, Trial 3, Inhibition (Executive Function). A similar post- LED result pattern was observed for the Domain Score for Learning and Memory. The LM Domain Score included 7 subtests: CVLT-II (total; short- and long- delay, free and cued recall); and the ROCFT (immediate and delayed recall). See Figure 4F.

#### Secondary Outcome Measures

**Figure 5:**
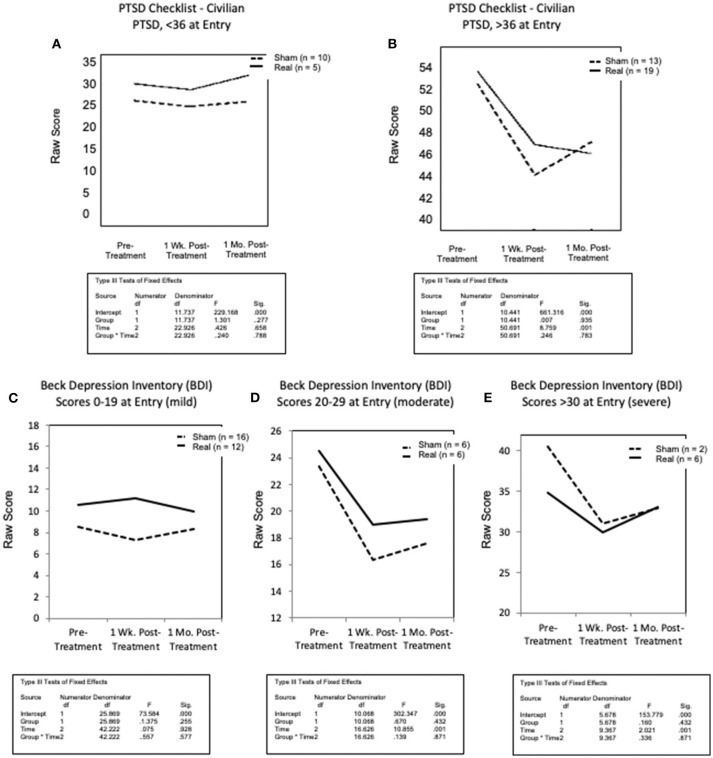
Graphed results for secondary analyses, for secondary outcome measures/psychosocial self-report/questionnaires for PTSD **(A,B)**; and depression **(C–E)**. All participants were studied for each secondary outcome measure, but only in subgroups as stratified by level of severity at entry [mild, moderate, severe]. **(A)** Scores less than 36 (mild or no PTSD) showed little change post-treatment in either group, or at either post- testing time point. **(B)** In contrast to that, for those entering with PCL-C scores greater than 36 (indicative of PTSD symptoms reported at the clinical level), both groups reported reduced PTSD at 1-week post-treatment. However, at 1-month, only the real LED group continued to report even fewer PTSD symptoms, while those in sham, reported increased symptoms, toward entry values. **(C)** For depression, on the beck depression inventory, there was no change for those with mild or no depression (BDI scores of only 0–19, at entry) in either treatment condition at any time of post- testing. **(D,E)** However, for those with moderate or severe depression (BDI scores of 20–29, or >30, respectively), those in the sham and real LED groups both reported less depression at 1 week post- treatment. At 1 month post- treatment, however, both groups began to return slightly toward entry values. No difference between sham and real LED was observed, likely due to small numbers in the subgroups.

Results for measures of PTSD and depression are shown in Figures 5A–E. Participants who had scores <36 on the PCL-C at Entry (mild symptomatology) showed little or no change over time in either the Sham LED or the Real LED groups (Figure 5A). However, those with scores >36 on the PCL-C at Entry showed the same pattern as that observed for the cognitive tests mentioned above – i.e., a reduction in PTSD at 1 week post- the Sham or the Real LED series, but at 1 month, only those who had received the Real LEDs continued to improve even more, with reduced PTSD scores at that time (Figure 5B). Those who had received the Sham LEDs regressed, toward PTSD Entry scores. One participant did not complete the PCL-C at Entry (declined to complete it).

On the BDI (Figures 5C–E), veterans in the mild group (scores of 0–19 at Entry) showed no change over time, in either treatment group. Veterans with moderate depression (scores 20–29), and severe depression (scores > 30) improved at 1 week Post- treatment, in both the Sham and the Real treatment groups. At 1 month Post- treatment, scores in both groups started back toward Entry, but did not return to Entry levels. Small numbers, with only 2 to 6 subjects in the moderate or severe, Sham or Real groups, likely affected the minimal group differences.

After all post- testing was completed, participants were provided the opportunity to comment on the study. They reported on psychosocial changes noticed outside the laboratory setting. A total of 10 participants offered comments, post- Sham or Real. There were 13 categories of comments from the Real LED group, and 6 categories of comments from the Sham LED group. Four participants who received Real, commented that they noticed increased concentration; one participant from Sham, mentioned increased concentration. See Table 5.

**Table 5:** List of open-ended comments/changes noticed by participants *outside* the laboratory setting, during or after the study.

	**No. of participants commenting in each category**	**Participant description of reported change**
**A) Real LED treatment series**		
Increased concentration	4	At work; at home; for everyday activities
Increased relaxation	4	
Improved sleep	3	Described as more, restful, better, good
“Helped/better” but could not elaborate	3	“Happy with treatment”
Increased work or social activity	2	Both participants report being previously withdrawn or not motivated. One returned to college, finished degree; then hired in new, Executive level position with a State Veterans Organization. One started group PTSD therapy, wellness and yoga, and felt he was able to be more social	
Tests/activities easier	2	
Decreased pain	2	Both decreased joint/muscle pain
Decrease in migraines	2	Both diagnosed with history of cluster migraines; these completely disappeared	
Improved gastro-intestinal problems	1	No longer stopped to use the bathroom several times on trip home
Improved cognition	1	
Decreased nightmares	1	Patient decided to reduce medications for nightmares/sleep (but this resulted in increased emotional lability); same participant as below	
Increased emotional lability	1	Occurred for about 2 weeks after reduction in nightmare medications	
Decreased anxiety	1	
**B) Sham LED treatment series**		
Increased activity	1	
Increased concentration	1	
Improved memory	1	Memory seems better
Improved focus	1	
Worse sleep	1	
Increased wakefulness	1	Attributed to naps during treatment	

*A total of 10/48 participants offered comments as categorized here*.

## Discussion

To our knowledge, this is the first Sham-controlled study to examine effects of tPBM therapy on cognition and health symptoms in veterans with GWI. In the Primary Analyses, which included data for all participants, results showed a significant effect in the Real LED group compared to Sham over time, for only one out of 17 NP tests, Digit Span Forwards (Working Memory, Executive Function) (*p* < 0.01), and a trend to significance for Trails 4, Number/Letter Sequencing (Executive Function) (*p* < 0.10).

Significant improvement was also observed in favor of Real compared to Sham, for only one secondary outcome measure SF-36V Plus, Physical Component Score (*p* < 0.08). Significant improvement on this test was previously reported in an acupuncture study with GWI veterans (65).

Under the null hypothesis we would expect false positives 5% of the time, a Type I error of 5%. This would yield around three false positives among 63 tests, including subtests. In fact, we observed three: (1) Digit Span Forwards; (2) Trails 4, Number/Letter Sequencing; (3) SF-36V Plus, Physical Component Score. Had we used a Bonferroni correction, then no results would have been significant and no implications could be drawn. Chance could not be ruled out. Thus, we performed Secondary Analyses to identify possible future predictors.

The Secondary Analyses were performed to include only participants for each NP test who scored below the norm (<50th percentile) at Entry, due to large heterogeneity in scores at Entry, consistent with literature describing the heterogeneity of GWI. A separate and distinct pattern of results was observed. When tested at 1 week after the final, 15th LED treatment, there was improvement on cognitive testing in both groups. When tested at 1 month, however, only those who had received Real, continued to show improvement, whereas those who received Sham, worsened with scores returning toward Entry value. Although, due to small numbers, there was no significant group x time interaction, five out of 17 NP tests showed improvement for the Real group at 1 month including: (1) Digit Span Total, Forwards and Backwards (Working Memory, Executive Function); (2) Trails 4, Number/Letter Sequencing (Executive Function); (3) CVLT, long delay, free recall (Verbal Memory); (4) CPT, False Alarm Rate (Sustained Attention); and (5) Color-Word Interference Test, Stroop, Trial 3, Inhibition (Executive Function).

A meta-analysis of GWI studies recommending which NP tests to use in GWI treatment efficacy studies (5) included the first four of the five NP tests mentioned above. That meta-analysis had also suggested a Visuospatial Memory Test, Block Design. Results from the NP improvements observed in the secondary analyses of this study suggest that the tests listed above may be useful as future predictors for inclusion in treatment efficacy studies, including for tPBM treatment studies.

Regarding the secondary outcome measures/Psychosocial Self-Report/Questionnaires in the Secondary Analyses, improvement occurred only when there was a higher level of symptomatology at Entry. When participants endorsed more PTSD symptomatology on the PCL-C (scores >36), there was reduced PTSD after both the Sham and Real LED series at 1 week. At 1 month, however, only those who had received Real, had additional improvement; whereas Sham worsened, toward Entry value. A somewhat similar pattern was present for the BDI. There was reduced depression for those with moderate or severe depression in both the Sham and Real groups at 1 week post- treatment, but slight increase in depression at 1 month post- treatment in both groups. Studies focusing on depression should include a greater number of subjects, even with moderate-severe depression, to better determine possible changes related to tPBM. Schiffer et al. (45), had reported a reduction in severe depression after only one tLED treatment to F3 and F4 (10–20 EEG system), however, after 1 month, the level of depression began to rise. Continued tPBM treatments may be necessary with depression.

Regarding a placebo effect, research in various treatment studies has shown there are three main components that can affect a placebo response (100): (1) assessment and observation, (2) therapeutic ritual, and (3) a supportive patient-practitioner relationship, with the latter being the most robust. The present study contained all three components, and in particular, the patient-practitioner relationship could have played a role in treatment response. There were bi-weekly interactions for at least a half hour, during each of the 15 treatment visits (Sham and Real), and this could have affected the 1-week post- testing. At the 1-month post- testing time point, however, there had been no bi-weekly visits for the previous 4 weeks. At the 1-month post- testing visit, scores in the Sham group declined toward Entry values, but scores in the Real group continued to improve, possibly separating out a placebo response that had occurred in the Sham group at 1 week.

An additional factor to consider regarding a placebo effect at 1-week Post- treatment, is the color green, which was visually perceived by all participants when wearing the goggles. Functional MRI research with migraine patients has reported that cortical hyperactivation associated with pain, can be reduced in migraine with tinted lenses (101); green light via cone-driven retinal pathways has been observed to have therapeutic effects (102, 103). The goggles may have indirectly had a beneficial, cortical effect for up to 1 week in the present study. This is unknown. The effect may have worn off at the 1-month post- testing time point resulting in a regression of scores toward Entry in the Sham LED group, whereas those in the Real LED group had scores that continued to improve. Two participants who received the Real LEDs commented after completion of the study that their migraine headaches had completely disappeared (Table 5). Since all subjects in each group wore the goggles, it is possible that the green color perceived through the goggles had a modulating effect on the cortical activation associated with pain in migraine, in accordance with the literature (101–103). In addition, the red photons from the red intranasal may have improved blood circulation, improving their migraines (63, 64).

Although small, the observed improvements following Real tPBM could have been associated with decreased inflammation (29), increased focal rCBF (45, 46), improved functional connectivity (51, 55, 57–59), as well as increased ATP production in hypoxic/stressed cells (26), all factors associated with PBM application.

This study was unique, in that it combined transcranial, intranasal, plus ear application of the LEDs to allow coverage of additional regions. While the relative contribution of any specific placement location of the LEDs is unknown, it is possible that a synergistic effect was obtained from all three. Specifics of LED placements need to be studied to understand how to obtain optimal effects. Future studies could also include blood work, and brain imaging studies such as rs-fcMRI, magnetic resonance spectroscopy, or arterial spin labeling to examine some of these factors.

Future tLED research could also include additional follow-up time points in order to better evaluate a long-term response. In the present study, the improvements observed in the Real LED group, which increased 1 month later, suggests a possible long-term effect of tPBM. A long-term pattern of improvement out to 2 months after a 6-week, Real tLED treatment series was observed in chronic, TBI/PTSD cases (49).

### Limitations

The sample size was small (48 cases), making it difficult to observe significant improvement, when considering the number of NP tests examined. Thus, chance cannot be ruled out. However, small effects were observed that may inform future studies. Although our study examined 17 NP tests in the Primary Analyses, our Secondary Analyses support testing GWI veterans, within three cognitive domains: attention/executive function, learning/memory, and visuospatial skills, which have been identified as areas of difficulty in GWI veterans (5). Including GWI veterans who perform poorly at Entry, in at least one or more of these three domains may be important in future studies, especially since there is large heterogeneity among the GWI veterans in cognitive abilities and symptomatology. This would improve the design, and statistical power, requiring fewer participants to determine significance.

In future tLED studies, using only NIR wavelengths (beyond the visible spectrum) and omitting any red LEDs, could avoid potential confound from goggles necessary to block visible red. Optimum tLED placement, treatment dose and treatment schedules are yet to be determined. In-office treatments at a VAMC (twice per week for almost 2 months) was difficult. An at-home tLED intervention would ease the burden of on-site visits, increasing enrollment and reducing dropouts. Treating at home could improve treatment compliance and allow for treating more often - e.g., three times per week (as in our previous TBI and stroke studies). In the present study, only 21% of those receiving Real tLED kept to the treatment schedule of twice per week (and 25% of Sham). Home treatments may also remove the patient-practitioner confounding interaction.

Technical improvements in PBM equipment since the time this study was designed (2012) have now made tLED home treatments feasible (57, 58, 66). The positive changes observed in the present study in cognition and reduced PTSD, support previous findings from the two case reports of GWI veterans treated at home with NIR tLED where symptom reduction was present in the mood-cognitive domain after 12 weeks of treatments, applied every other day (66). That study also observed reduced pain, sleep and fatigue; these improvements may have been associated with more frequent treatments (three times per week) and a longer treatment period (12 weeks), compared to the present study. Weekly telehealth phone calls could establish treatment compliance.

## Conclusions

This study was underpowered (*n* = 48), with large heterogeneity at Entry, which likely contributed to significance or trend to significance, for only two of the NP tests (Digit Span Forwards; and Trails 4, Number/Letter Sequencing) and the SF-36V Plus, Physical Component Score. GWI veterans who exhibit cognitive impairments, particularly in the domains of Executive Function, Working Memory, Sustained Attention and/or Verbal Memory are likely good candidates for future tPBM studies. Transcranial PBM is a safe therapy without negative side effects or adverse events. Controlled studies with newer, transcranial LED home treatment devices are warranted; this is expected to increase enrollment.

## Data Availability Statement

The datasets presented in this article are not readily available because Datasets are available by written request to the PI, MN, with approval by the VA Boston Healthcare System IRB, Privacy and Security Offices. Requests to access the datasets should be directed to MN, mnaeser@bu.edu.

## Ethics Statement

The studies involving human participants were reviewed and approved by The Institutional Review Board at the VA Boston Healthcare System and the San Francisco VA Medical Center (University of California, San Francisco). In accordance with the Declaration of Helsinki, Informed Consent and HIPAA authorization were obtained. The VA Boston Healthcare System, Safety Subcommittee of the IRB approved all LED devices. The patients/participants provided their written informed consent to participate in this study. Written informed consent was obtained from the individual(s) for the publication of any potentially identifiable images or data included in this article.

## Author Contributions

PM: study coordinator, study design and implementation, recruitment and screening, data management, collection, scoring, statistics, and manuscript writing/preparation. MN: Principal Investigator (PI)- Boston, study design and implementation, study oversight, manuscript writing/preparation, PBM oversight, and training. LC: PI- San Francisco, study implementation and oversight (SF), and manuscript preparation. MK: study design and implementation, neuropsychologist, data collection, and manuscript preparation. MDH: study design and implementation, treatment application, and manuscript preparation. MY: recruitment, screening, data collection, scoring, and statistics. RL: statistics. JK: neuropsychologist, study design, data collection, and manuscript writing/preparation. MRH: PBM expertise/safety consultation and study design. All authors contributed to the article and approved the submitted version.

## Conflict of Interest

MRH declares the following potential conflicts of interest: Scientific Advisory Boards: Transdermal Cap Inc, Cleveland, OH; BeWell Global Inc, Wan Chai, Hong Kong; Hologenix Inc. Santa Monica, CA; LumiThera Inc, Poulsbo, WA; Vielight, Toronto, Canada; Bright Photomedicine, São Paulo, Brazil; Quantum Dynamics LLC, Cambridge, MA; Global Photon Inc, Bee Cave, TX; Medical Coherence, Boston MA; NeuroThera, Newark DE; JOOVV Inc, Minneapolis-St. Paul MN; AIRx Medical, Pleasanton CA; FIR Industries, Inc. Ramsey, NJ; UVLRx Therapeutics, Oldsmar, FL; Ultralux UV Inc, Lansing MI; Illumiheal & Petthera, Shoreline, WA; MB Lasertherapy, Houston, TX; ARRC LED, San Clemente, CA; Varuna Biomedical Corp. Incline Village, NV; Niraxx Light Therapeutics, Inc, Boston, MA. Consulting; Lexington Int, Boca Raton, FL; USHIO Corp, Japan; Merck KGaA, Darmstadt, Germany; Philips Electronics Nederland B.V. Eindhoven, Netherlands; Johnson & Johnson Inc, Philadelphia, PA; Sanofi-Aventis Deutschland GmbH, Frankfurt am Main, Germany. Stockholdings: Global Photon Inc, Bee Cave, TX; Mitonix, Newark, DE. The remaining authors declare that the research was conducted in the absence of any commercial or financial relationships that could be construed as a potential conflict of interest.
